# Preparation of Oxygenated Water and Its Therapeutical Use

**Published:** 1863-03

**Authors:** 


### PREPARATION OF OXYGENATED WATER AND ITS THERAPEUTICAL USE.

Dr. Ozanam gives the name of oxygenated water to water which is distilled and afterwards charged with oxygen under the influence of high pressure. The experiments he has made have led him to establish three modes of operation by this new medicine. 1. It improves the condition of the blood in cases where that fluid is impaired or deficient, as in dyspnœa, asthma, slow asphyxia, cyanosis, diseases of the heart, hæmorrhoids, and hæmorrhoidal visceral congestion. 2. It possesses an oxidizing or metamorphic action in cases where the organic products are arrested in their development, as happens in glycosuria, gout, the uric and oxalic gravel, and perhaps in scrofula. 3. It exerts an exciting and regulating action on the brain and the thyroid gland, and hence its use in goitre and cretinism. If, in fact, snow-water taken as drink gradually produces these morbid conditions, it is because it is entirely deprived of vital air. On the other hand, oxygenated water, as well as the inhalation of gaseous oxygen, produces no results in hemierania, and unfavorable ones in cases of inflammatory disease. Thus, in croup, the oxygen temporarily tranquilizes the dyspnœa, but it increases the fever. In the treatment of ulcerated cancer the oxygenated water revives pretty well the powers of the patient, and the wounds assume a more vivid and rosy color, but they do not heal; and if the surfaces are bathed with rags steeped in oxygenated water, even when very slightly charged, the ulcer is soon observed to become gangrenous on the surface. Oxygenated water is perfectly limpid and pure, and the gas is disengaged in the form of very fine bubbles. Having little taste, it resembles in this respect water which is deprived of air; and, like the latter, it is a little heavy for the stomach.—*B. and F. Med.-Chir. Rev.*, July, 1862, from *Comptes Rendus de l' Acad. des Sc.*, November, 1862.

